# Parents' perceptions of physical activity for their children with cancer: a qualitative meta-synthesis

**DOI:** 10.3389/fped.2025.1402516

**Published:** 2025-03-28

**Authors:** Yan Gu, Jie Yu, Liangjian Li, Liming Pan, Dongmei Ma, Lin Cui, Chunxue Cui, Fang Guo

**Affiliations:** ^1^ Department of Cardiovascular Disease Center, The First Hospital of Jilin University, Changchun, China; ^2^ Department of Pediatric Hematology, Children's Medical Center, The First Hospital of Jilin University, Changchun, China; ^3^ Department of Hand and Podiatric Surgery, Orthopedics Center, The First Hospital of Jilin University, Changchun, China; ^4^ Department of Nursing, The First Hospital of Jilin University, Changchun, China; ^5^ School of Nursing, Changchun Medical College, Changchun, China

**Keywords:** children, cancer, physical activity, qualitative research, systematic review

## Abstract

**Background:**

The the number of cases of cancer in children is increasing annually. Physical activity (PA) can enhance the future outcomes and quality of life of children with cancer. However, studies have shown that children with cancer have low levels of PA and that the majority don't adhere to the guidelines' recommendations for physical activity.

**Objective:**

The aim was to synthesize parents' perceptions of PA for their children with cancer and to explore barriers and facilitators, thereby providing a basis for promoting PA in children with cancer.

**Methods:**

The PubMed, Web of Science, Cochrane Library, EMBASE, CINAHL and three Chinese databases were systematically searched. Qualitative empirical reports from the onset of the corresponding databases until October 2023 were included in the review. Two independent reviewers performed the review, carried out the data extraction process and evaluated the study quality.

**Findings:**

Six studies in all were included. Parents’ perceptions of PA for their children with cancer were synthesized into the following three themes of analysis: (1) barriers to participation in PA; (2) facilitators of participation in PA; and (3) seeking support.

**Conclusion:**

Our study identified complex factors that influence physical activity participation in children with cancer, and these findings provide a focus for future interventions. Future research should further explore appropriate, targeted exercise intervention programs to promote children's participation in physical activity.

## 1 Introduction

Cancer is one of the leading causes of death among children and adolescents worldwide. The data show that the overall incidence of cancer in children and adolescents is increasing at a rate of 0.8% per year (1). However, with advances in cancer treatment and management, the 5-year survival rate of children with cancer has increased to 85%, and for adolescents, it has increased to 86%. This means that the number of child and adolescent cancer survivors is increasing and is currently estimated to be more than 5 million people (1, 2). Unfortunately, survival is not without increased health risks, and health risks increase with age. A lifetime cohort study at St. Jude Hospital reported that approximately 95.2% of childhood cancer survivors experienced delayed effects after treatment, with survivors experiencing twice the disease burden of healthy controls by 45 years of age (3). The most common delayed effects include fatigue, neurocognitive deficits, cardiovascular disease, and skeletal and reproductive system problems, leading to a reduced quality of life (4, 5). As a result, many researchers are taking action to identify complementary therapies that can reduce cancer side effects and conventional cancer treatments.

Physical activity (PA) is a health behavior that prevents and mitigates adverse outcomes from cancer and its treatment. In recent years, a growing number of academics have noted the positive impact of promoting PA on child survivors. There is strong evidence to support that regular PA can lessen the negative side effects of treatment that children who have survived cancer must endure, such as potentially promoting bone development and reducing metabolic syndrome and cardiovascular disease risk (6). Unfortunately, PA levels among childhood cancer survivors are decreasing childhood cancer survivors are not considered active enough compared to their healthy peers, with 52.1% of survivors not meeting the PA guideline recommendations (7). A study of PA practices among childhood cancer survivors showed that 37.0% did not engage in regular PA (8). In addition, childhood cancer survivors reported that the side effects of treatment medications result in a decline in health status that hinders participation and development of PA, making it difficult to return to pre-diagnosis levels for a significant period of time (9, 10).

An increasing amount of research is focusing on the nature of PA in childhood cancer survivors, uncovering facilitators of and barriers to engaging in PA to improve survivors' PA levels. A systematic evaluation revealed that perceived competence and concerns about the body prevented survivors from engaging in PA, while parental and social support motivated them to participate (11). Unsurprisingly, parental support and encouragement are important factors in improving PA in children and adolescents (12). Therefore, there is a need to explore parents' perceptions of PA for their children with cancer to promote and develop PA in child cancer survivors. Multiple qualitative studies have been carried out in several countries, but no meta-analysis of how parents view PA in their cancer-stricken children has been done.

Therefore, this review was carried out in order to identify and synthesize parents' perceptions of PA for their children with cancer and to analyze the barriers to and facilitators of PA participation for children with cancer, providing a foundation for the development of intervention programs customized to meet individual requirement. This review was driven by the following question: (1) What are parents' perceptions (barriers and facilitators) of PA for their children with cancer? (2) What recommendations about PA can be conducted regarding further clinical practice, research, and education based on the included studies?

## 2 Review

### 2.1 Design

Qualitative meta-synthesis is used to integrate qualitative research exploring the same or related topics to create a more comprehensive understanding of the phenomenon and increase the validity of the findings (13).

This review was designed as a qualitative evidence synthesis using thematic synthesis (14) and was conducted in accordance with the guidelines for Enhancing Transparency in Reporting Qualitative Research Synthesis Reports(ENTREQ) (15) (Supplementary Appendix S2).

### 2.2 Inclusion and exclusion criteria

Either Chinese or English-language publications were be included. To be considered for this review, all studies had to satisfy the PICOS (P = participant, I = phenomenon of interest, Co = context, and S = type of study) (Table 1). Analysis and extraction of mixed-method studies were limited to their qualitative components.

**Table 1:** Inclusion and exclusion criteria for study selection.

Criteria	Inclusion criteria	Exclusion criteria
Participants	Parents of children with cancer	Other caregivers of children with cancer
Phenomenon of interest	Views on PA	The study not express parents percetions of PA for their children surviving cancer
Context	Primary, secondary and tertiary health care backgrounds	
Types of studies	Qualitativa components of qualitative and mixed methods research	Quantitative study

### 2.3 Search strategy

For publications published in either English or Chinese from the inception to October 2023, the five English databases (Web of Science, PubMed, CINAHL, Embase and Cochrane Library) and three Chinese databases (CNKI, VIP and Wanfang Data) were be searched. The search terms were “Neoplasms”, “Tumor”, “Cancer”, “Child”, “Adolescent”, “Parent”, “Mother”, “Exercise”, “Physical Activity”, “Sports” and “Qualitative Research”. The search strategy for each database was detailed in Supplementary Appendix S1. Furthermore, a manual search was conducted through the reference lists of the included studies and systematic reviews to guarantee the retrival of all pertinent studies.

### 2.4 Study selection

The selection of studies followed the Preferred Reporting Items for Systematic Reviews and Meta-Analyses (PRISMA) guidelines. The PRISMA flowchart covers the selection process for the inclusion of studies (16). Endnote 20 reference management software was used to manage the search items. After deleting duplicate records, the study titles and abstracts were separately checked by two reviewers, who then read each study for further assessment. Disagreements amongst reviewers regarding eligibility were resolved through discussion with a third reviewer.

### 2.5 Quality appraisal

To ensure the quality of the studies assessed, two reviewers independently assessed the included studies using the JBI qualitative assessment tool and discussed the results of their assessments (17). When the assessments were inconsistent, the quality of the included studies was first discussed and, if necessary, decided by a third reviewer. The aim of the qualitative assessment was to emphasize the quality of the evidence on the subject based on a systematic and standardized process, rather than to exclude poor quality studies. Therefore, studies were not excluded due to quality bias.

### 2.6 Data extraction and analysis

Data extraction was conducted independently by two researchers. The following characteristics was specifically extracted from the included studies: author, year, country, aim, methodology, participants and findings (primary themes, subthemes, or key findings).

Qualitative data were analyzed using Thomas and Harden's thematic analysis (14). In the first phase, two reviewers independently coded all descriptions related to parents’ experiences and perceptions of PA for their children with cancer. In the second stage, reviewers grouped to develop new codes to arrange descriptive themes after comparing and contrasting existing codes. The descriptive themes from the previous stage were continually reviewed and analyzed in the third phase to produce analytical themes that explained all of the descriptive themes as well as inferred experiences and perceptions.

### 2.7 Assessment of confidence in the review findings

JBI's ConQual system was used to assess the confidence level of synthesized evidence from qualitative studies and to rate evidence reliability and credibility (18). All meta-synthesized evidence was assumed to be of high quality and subsequently assessed according to the three dimensions of credibility and the five dimensions of reliability, resulting in a high, medium, low or very low quality rating.

## 3 Results

### 3.1 Search outcomes

**Figure 1:**
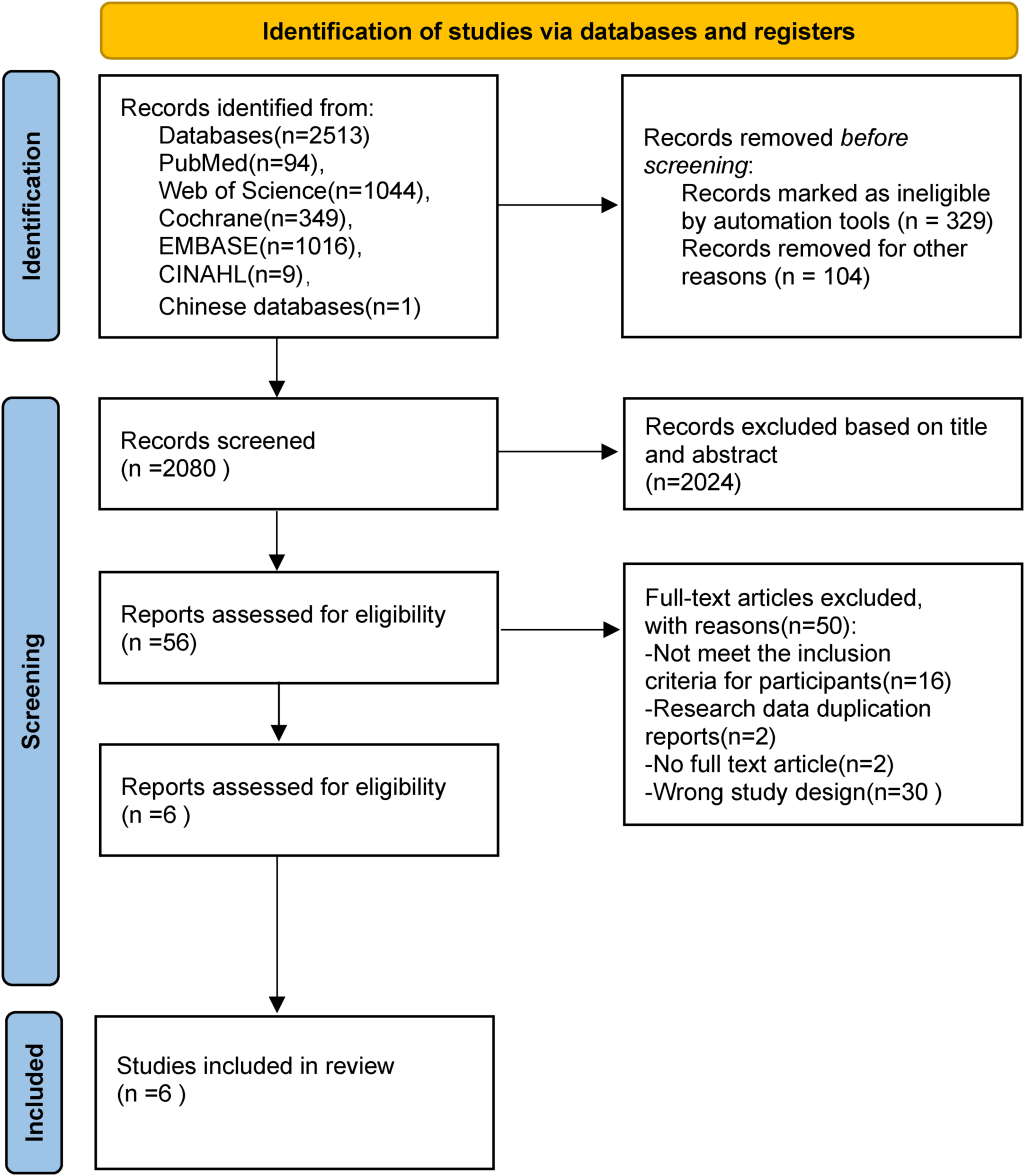
Flow diagram of the search for and selection of the included studies.

Following a preliminary database search that produced 2,513 publications, 56 studies were kept for additional assessment following the removal of duplicates and the screening of titles and abstracts. Six qualitative studies in all were eventually included, five published in English and one in Chinese (Figure 1). The search of the reference lists yielded no more referances.

### 3.2 Characteristics of included studies

These studies were conducted in Norway, Denmark, Singapore, Australia and China. Qualitative methods used included thematic analysis (*n* = 4) and phenomenological analysis (*n* = 2), and data were collected through semistructured interviews. A total of 153 parents were included (Table 2).

**Table 2:** Characteristics of the study.

Author/year/country	Aim	Qualitative approach	Participants	Key findings/themes
Larsen et al. (19) 2023 Norway	To identify perceived barriers and facilitators to PA in young survivors and their parents.	Descriptive research; thematic analysis method	Childhood cancer Survivors (*n* = 63) Parents (*n* = 68)	Barriers to physical activity; Moderating factors of PA participation
Petersen et al. (24) 2022 Danish	To investigate how parents and children who have survived pediatric cancer experience a combined physical and social activity intervention during treatment, as well as how they view physical activity after treatment.	Descriptive research; thematic analysis method	Childhood cancer Survivors (*n* = 18) Parents (*n* = 19)	Being physically active during hospitalization; Peers as motivators; Physical activity post-­treatment
Weller et al. (20) 2023 Singapore	To investigate how PCCS is perceived in Singapore and what potential role they might play in PA.	Semi-structured interviews; thematic analysis method	Parents (*n* = 7)	The barriers and enablers of PA; Impact of cancer
Grimshaw et al. (21) 2021 Australia	To investigate how parents view physical activity for kids during acute cancer treatment and what tactics they think could assist kids become more physically fit.	A constructivist perspective; thematic analysis method	Parents (*n* = 20)	Factors that contribute to physical inactivity; Parental response to physical inactivity; Parental perspectives on overcoming physical in activity
Cheung et al. (23) 2021 Hong Kong	To gain insight into how parents view their children's physical activity and figure out what encourages and hinders engagement in physical activity for kids with cancer.	Descriptive phenomenological approach	Parents (*n* = 28)	Perception of physical activity; Awareness of their child's physical activity level; Perceived barriers; Perceived facilitators of performing physical activity for their children.
Ouyang et al. (22) 2019 China	To explore the barriers of physical activity for children with malignant tumor during treatment	Semi-structured interviews; phenomenological research	Childhood cancer Survivors (*n* = 6) Parents (*n* = 11)	Disease and Treatment Related Factors Traditional cognitive factors Psychological and Socio-environmental factors

### 3.3 Methodological quality

Each study satisfied at least 6 criteria points, and only 1 study received a score of 8 points. However, the representativeness and typicality of the research population of the four studies were not especially stellar. In addition, Questions6 and 7 were not answered for these studies because precise details regarding the researchers' cultural background and the relationship between the study and researchers was not provided (Table 3).

**Table 3:** Results of the critical appraisal of the studies included.

	Q1	Q2	Q3	Q4	Q5	Q6	Q7	Q8	Q9	Q10
Larsen et al. (19)	Y	Y	Y	N	Y	N	N	U	Y	Y
Petersen et al. (24)	Y	Y	Y	N	Y	N	U	Y	Y	Y
Weller et al. (20)	Y	Y	Y	Y	Y	N	N	Y	Y	Y
Grimshaw et al. (21)	Y	Y	Y	N	Y	N	N	Y	U	Y
Cheung et al. (23)	Y	Y	Y	N	Y	N	N	Y	Y	Y
Ouyang et al. (22)	Y	Y	Y	Y	Y	N	N	Y	Y	N

### 3.4 Confidence in the findings

The confidence of individual review results was assessed based on the ConQual system. The evidence for each level in the qualitative study was clear, so the confidence level remained the same. However, due to reliability constraints, the findings were lowered by 1 level. Consequently, the ConQual level was judged to be of medium confidence (Table 4).

**Table 4:** Summary of findings and ConQual assessments.

Synthesized findings	Type	Dependability	Credibility	ConQual score
Barriers to PA participation	Qualitative	Downgrade one level^a^	Remains unchanged^b^	Moderate
Facilitators of PA participation	Qualitative	Downgrade one level^a^	Remains unchanged^b^	Moderate
Seeking support	Qualitative	Downgrade one level^a^	Remains unchanged^b^	Moderate

^a^ Downgraded 1 level because of common dependability issues across the included primary studies (most studies had no statement locating the researcher and no acknowledgment of their influence on the research).

^b^ Remains unchanged as all findings unequivocal.

### 3.5 Qualitative synthesis

The meta-synthesis identified three themes and eight subthemes from the extracted data. Parents' perceptions of PA for their children with cancer were categorized into three themes: (1) barriers to participation in PA; (2) facilitators of participation in PA; and (3) seeking support.

#### 3.5.1 Barriers to PA participation

##### 3.5.1.1 Physical limitations

In this theme, parents reported impaired body structure and function due to cancer treatment and delayed effects as the primary barrier to their children's inability to engage in PA. Parents reported that cancer treatment was devastating for their children, and physical fatigue from cancer and its treatment was thought to limit their children's ability to participate in PA (e.g., playing with peers, going to school): “*He is very affected by the fact that he had that disease and has been put very back. His is weak in terms of endurance and muscles. And his coordination has been bad”* (19). *“The chemo can make you fatigue, some got very strong side effect, vomiting, high blood pressure… they limit the PA. His friends, you cannot say they are lazy, because of the lung function and they feel tired”* (20). “*…he's lost muscle mass and lost his fitness, lost the weight, everything…he gets really tired, his fitness, his energy level, his food intake, it's all changed”* (21). Neurological delays, fatigue, and reduced muscle strength contribute to the children's decreased endurance, coordination, and motor skills, which impeded their participation in PA.

##### 3.5.1.2 Negative parental attitudes toward PA

For most of the parents, performing PA was not a priority compared to cancer treatment and recovery, so they ignored the benefits of PA for their children and thus did not encourage PA. Second, influenced by traditional cultural thinking, parents believed that rest and recuperation were more conducive to their children's physical recovery: *“when she goes out for exercise, how little she consumes energy and harms yang, and the balance between yin and yang is the best”* (22). In addition, the parents had some concerns about their children's participation in PA; they recognized that the effects of cancer had weakened their children and therefore limited or discouraged their children from participating in PA to protect their children from harm, especially if the activity did not ameliorate the effects of the cancer: *“I’m afraid that my daughter may get hurt when performing physical activity as she is not as physically fit as before. So, to play safe, I ask her to avoid doing vigorous exercises”* (23). “*I didn't want him to move from the bed at the beginning… because I’d seen him kind of dying and I was like just keep him there until he gets better”* (21). Additionally, parents reported caring for their children with cancer to be a significant emotional challenge. Parents reported that they were exhausted by the cancer experience and were in perpetual fear of cancer recurrence, and that these adverse emotions hindered their ability to take on anything else: *“…you’re in so much shock and the child is so young and they’re so distressed and there's just so much mental stuff going on that I don't think we thought about it unless, you know, the doctors or someone would say something about physical activity”* (21).

##### 3.5.1.3 Psychological disorders

Personality changes and social isolation were among the factors that limited the children's participation in PA. Parents described issues of declining mental health, such as isolation, fear, and loss of self-confidence, as a result of their children's prolonged social isolation due to the disease, further leading to reclusive behaviors and limiting PA participation: *“after she got sick, she didn't go out and get in touch with other people. She just held iPad alone. I think she was very lonely.”* (22). “*…he says he hateswhat he sees in themirror andhe just won't let anyone, apart from family see him”* (21).

##### 3.5.1.4 Insufficient social support

Parents identified the lack of specialized health care providers as another barrier to PA participation. Both the parents and their children need professional guidance, and this barrier could be a possible facilitator if health care providers were supportive*: “I think they [the physical activity professionals] could have pushed her more. “They didn't need to leave it up to them [the children] to [decide to] participate. They could have just told her it was a part of being in RESPECT”* (24).

##### 3.5.1.5 Logistical obstacles

For families, the integration of PA into daily life is challenging and can be limited by many factors such as time, environment, and policies. Parents described challenges in implementing PA due to hospital factors, such as the hospital environment, facilities, and policies limiting their children's ability to engage in PA: *“When she's on the ward, no, zero activity, what do you do apart from…walk your drip to the hub. They do nothing, they can't, and there's nothing really they can do”* (21). Several environmental features, including complex transportation, scarce recreational facilities, and a lack of sports activities, hinded this process: *“What can you do in the Singapore context right?”* (20). In addition, time delivery difficulties were also a limiting factor reported by parents, as their children struggled to find time for PA due to academic issues and the parents lacked the time and energy to be physically active with their children due to work: *“I have to go to work during the day….At weekends, I just want to take a rest at home, I can't think of doing any physical activity with my daughter.” “My kid has loads of homework and revision to do every day. It seems that she doesn't have spare time for doing physical activity”* (23).

#### 3.5.2 Facilitators of PA participation

##### 3.5.2.1 Family support

Parental support and encouragement influence children's moods and motivation to participate in PA, especially when parents perceive PA benefits. Most parents recognized the positive impact of PA on improving their children's overall conditions, so they supported and encouraged their children's participation in PA. Some parents reported that when they were with their child, the child showed a strong interest in participating in PA: *“If his dad goes out for physical activity, my son will follow”* (23). *“We decided that we could not both work full time, our son needed a parent facilitating at home.”* (19). In addition, parents described other facilitators such as sibling companionship and family activity styles: *“The trampoline and the daily competitions with his active brother helped him getting faster back his balance and his motor skills”* (19).

##### 3.5.2.2 Individual factors

Children with cancer may face numerous challenges due to individual differences in age, individual traits, personality type, and interests: *“Because he's a boy… yeah, I don't know if this is a boy thing,or just him? He's very active”* (20). In different studies, parents expressed the importance of PA design and selection regarding their children's interests and preferences: *“It's really important to know what types of sports my child is interested in as performing the sports that he is interested in can allow him to enjoy sports”* (23).

##### 3.5.2.3 Peer support

Bonding with peers is recognized as a facilitator of PA. Most parents reported that competition motivated their children to reach the PA level of their peers and promoted socialization enhancing their children's social skills:*“[…], that they always did a bit more than he could. He was eager to come in first place every time, so he pushed himself to do the same exercises as they did”* (24).

#### 3.5.3 Seeking support

Parents across the different studies expressed their desire for access to professional support and guidance. There was a general need for parents to obtain specialized knowledge about PA, such as how to exercise, and how much to exercise: *“I have heard that there is a recommendation for the desirable amount of physical activity for children, but I don't know the exact amount of it”* (23). Furthermore, parents expressed a desire for environmental support. Parents reported wanting the hospital to be more supportive of the planning and design of the environment, facilities, and policies that would be conducive to PA participation for children with cancer: *“If they have a small gym, yeah will be quite helpful”* (20). As previously stated, both parents and children experienced great psychological challenges after a cancer diagnosis, and parents want professional psychological support. In addition to this, parents described the need to communicate with other parents in similar situations as they believed that parent-to-parent support was helpful: *“… then I really, really,really need to talk to people in the same situation because, … I had to introduce myself, I said, like, “oh, you know, my child here, he has this very similar to your situation, can we start a chat?”* (20).

## 4 Discussion

This qualitative synthesis synthesized existing evidence on parents' perceptions and experiences of PA involvement for their children with cancer. Three themes were constructed: facilitators of PA participation, barriers to PA participation, and support seeking. These results provide insights for promoting participation in a purposeful and planned PA program for children with cancer.

By synthesizing published data, we found that the most common barrier to declining PA levels in most children with cancer is the impairment of physical function due to disease or treatment side effects. Although the cure rate for cancer has increased to 85%, there are persistent effects of cancer and long-term anticancer treatments on children, such as cardiorespiratory health and cancer-related fatigue (25). Studies have shown that cancer-related fatigue is the most prevalent aftereffect in children with cancer (26). Fatigue causes children to feel physically exhausted and to have reduced functional capacity and endurance, causing them to lose motivation to engage in PA. As reported by parents, fatigue is the main reason for decreased exercise in children. In fact, PA has a positive impact on improving children's physical functioning. A meta-analysis showed that PA had the most pronounced beneficial effects on fatigue, muscle strength, bone development, and cardiorespiratory health (27). However, most children and parents ignore this effect and limit PA participation.

Our study suggested that negative parental attitudes toward PA are another barrier to their child's participation in PA. It was found that after the diagnosis of cancer in their child, treatment and recovery were parents' main concerns, and the large amount of new information and complex treatments caused them to experience a heavy caregiving burden and psychological stress (28). This reason leads to a decrease in parents' ability to take on other things, and they ignore the positive effects of PA. Social cognitive theory suggests that cognition is an important factor influencing individual behavior (29). Due to social and cultural influences, parents' cognition of PA is biased and incorrect. Most parents believe that the balance between yin and yang is the key to maintaining good health, and that PA can disrupt this balance, leading to debilitation, or even worsening it. In order to prevent such events from occuring, parents restrict their children's participation in PA. In contrast, many studies have proved that PA can improve children's debilitation and enhance their health (27, 30, 31). Therefore, there is a need to change parents' misperceptions, and education on health information such as PA, is recommended to be added to future interventions to promote children's participation in PA.

The findings also suggested that psychological barriers, inadequate social support, etc. are also challenges to PA participation for children and parents. As a result of cancer, many children developd negative emotions such as depression and isolation (32), which prevent them from focusing on their own health. Therefore, healthcare workers should pay attention to the psychological changes of experienced by children and provide professional psychological support as an initiative to address psychological disorders. Inadequate social support, such as a lack of specialized healthcare providers, was likewise identified as a hindering factor, implying that accurate information and guidance from professionals could alleviate parents' and children's anxiety and concerns about PA. In addition, logistical barriers such as a lack of time, geographic location, and a lack of facilities were reported. This finding suggested that PA program organizers should consider intervention design preferences and mobilize and allocate more resources to provide practical PA programs.

By synthesizing the qualitative data, we identified numerous push factors that directly or indirectly contribute to a child's participation in PA. At the individual level, a child's perceptual-motor ability, intrinsic motivation, and preference for PA determine their willingness to participate in PA. Self-determination theory suggests that intrinsic motivation inspires behavior, and that intrinsic motivation is determined by an individual's positive perception of the external environment (33). Indeed, honoring a child's PA preferences can facilitate these positive perceptions. Therefore, PA programs should fully consider that children's PA needs to be tailored to support the promotion of positive experiences. Importantly, personal factors such as age, gender, and personality type should also be considered. Social support from family, peers and school plays an important role in improving children's PA. Research has shown that parental support and encouragement can promote children's participation in PA. Moreover, parents' positive perceptions of participation in PA have a positive impact on shaping children's PA beliefs and behaviors. In addition to parents, the presence and support of other family members are equally important. Future research could develop family-based interventions to increase PA-related education and enhance family motivation. On the other hand, understanding and support from peers and schools can help children escape social isolation and promote more positive PA experiences.

### 4.1 Limitations

First, six published qualitative studies were included in this review; no search of the gray literature was done. As a result, this review may not provide a full view of parents' perceptions and experiences of PA participation for their children with cancer. Second, due to language limitations, the review only searched for articles published in English and Chinese; therefore, important evidence published in other languages may be missing. Furthermore, the majority of the research was carried out in developed countries, where cultural, political, and environmental variations may impact how broadly applicable the current findings are. Finally, due to methodological limitations, the included studies may affect the generalization of the current findings.

## 5 Conclusion

Thus far as we are aware, this study is the first to synthesize parents' perceptions and experiences of PA for their children with cancer and to identify barriers to and facilitators of PA participation in children with cancer. Based on our results, children with cancer still face many challenges in participating in PA, the factors influencing children's participation in exercise are complex, and future research should further explore individualized exercise intervention programs to increase the willingness of children with cancer to participate in PA. In addition, family involvement, social support, and personal factors are critical for promoting children's participation in PA, and we expect future research to incorporate these facilitators to provide more diverse exercise programs to promote PA.

## 6 Relevance to clinical practice and research

This study explores barriers and facilitators to PA in children with cancer from the parents' perspective in order to promote regular PA participation. In clinical practice, there is a need for healthcare providers to provide targeted education to parents of children with cancer to improve parental perceptions of PA, increase their perceived benefits of PA, and further promote participation in and maintenance of PA for children with cancer. Future research should consider the investment of resource facilities to provide diversified and practical support for children with cancer. In addition, the results of the study may help researchers and healthcare providers to design comprehensive, individualized research protocols to increase willingness of children with cancer to participate in PA and ensure exercise adherence.

## Data Availability

The original contributions presented in the study are included in the article/Supplementary Material, further inquiries can be directed to the corresponding author.
