# Supplementary material for: Parents' perceptions of physical activity for their children with cancer: a qualitative meta-synthesis
Source: Front Pediatr. 2025 Mar 28;13:1402516. doi: 10.3389/fped.2025.1402516 (PMC11985796; doi:10.3389/fped.2025.1402516)
Supplement: Supplementary file 1 [file Table1.docx]

| Pubmed | | |
| --- | --- | --- |
| #1 | "Neoplasms"[Mesh] | 387,863 |
| #2 | Tumor[Title/Abstract] OR Neoplasm[Title/Abstract] OR Neoplasia[Title/Abstract] OR Cancer[Title/Abstract] OR "Malignant Neoplasm"[Title/Abstract] OR Malignancy[Title/Abstract] OR "Malignant Neoplasms"[Title/Abstract] OR "Neoplasm, Malignant"[Title/Abstract] OR "Neoplasms, Malignan"[Title/Abstract] OR leukemia*[Title/Abstract] OR “Hematologic Neoplasm”[Title/Abstract] OR “Neoplasm, Hematologic”[Title/Abstract] OR  “Hematologic Malignancy”[Title/Abstract] OR “Hematological Neoplasm”[Title/Abstract] OR “Neoplasm, Hematological”[Title/Abstract] OR “Malignancy, Hematologic”[Title/Abstract] OR “Blood Cancer”[Title/Abstract] OR “Cancer, Blood”[Title/Abstract] OR “Hematological Malignancy”[Title/Abstract] OR “Malignancy, Hematologica”l[Title/Abstract] OR “Hematopoietic Neoplasm”[Title/Abstract] OR “Neoplasm, Hematopoietic”[Title/Abstract] OR “Hematopoietic Malignancy” | 86,780 |
| #3 | #1 OR #2 (Neoplasms[MeSH Terms]) OR (Tumor[Title/Abstract] OR Neoplasm[Title/Abstract] OR Neoplasia[Title/Abstract] OR Cancer[Title/Abstract] OR "Malignant Neoplasm"[Title/Abstract] OR Malignancy[Title/Abstract] OR "Malignant Neoplasms"[Title/Abstract] OR "Neoplasm, Malignant"[Title/Abstract] OR "Neoplasms, Malignan"[Title/Abstract] OR leukemia*[Title/Abstract] OR "Hematologic Neoplasm"[Title/Abstract] OR "Neoplasm, Hematologic"[Title/Abstract] OR "Hematologic Malignancy"[Title/Abstract] OR "Hematological Neoplasm"[Title/Abstract] OR "Neoplasm, Hematological"[Title/Abstract] OR "Malignancy, Hematologic"[Title/Abstract] OR "Blood Cancer"[Title/Abstract] OR "Cancer, Blood"[Title/Abstract] OR "Hematological Malignancy"[Title/Abstract] OR "Malignancy, Hematologica"l[Title/Abstract] OR "Hematopoietic Neoplasm"[Title/Abstract] OR "Neoplasm, Hematopoietic"[Title/Abstract] OR "Hematopoietic Malignancy"[Title/Abstract]) | 391,564 |
| #4 | ("Child"[Mesh]) OR "Child, Preschool"[Mesh] OR "Adolescent"[Mesh] | 337,907 |
| #5 | Children[Title/Abstract] OR “Preschool Child”[Title/Abstract] OR “Children Preschool”[Title/Abstract] OR “Preschool Children”[Title/Abstract] OR OR Adolescent*[Title/Abstract] OR Adolescence[Title/Abstract] OR Teen[Title/Abstract] OR Teenager[Title/Abstract] OR Youth[Title/Abstract] | 155,899 |
| #6 | (Children[Title/Abstract] OR "Preschool Child"[Title/Abstract] OR "Children Preschool"[Title/Abstract] OR "Preschool Children"[Title/Abstract] OR OR Adolescent*[Title/Abstract] OR Adolescence[Title/Abstract] OR Teen[Title/Abstract] OR Teenager[Title/Abstract] OR Youth[Title/Abstract]) OR (("Child"[Mesh]) OR "Child, Preschool"[Mesh] OR "Adolescent"[Mesh]) | 376,860 |
| #7 | parent[Title/Abstract] OR cargiver[Title/Abstract] OR Family[Title/Abstract] OR father[Title/Abstract] OR mother[Title/Abstract] | 124,740 |
| #8 | ((((("Exercise"[Mesh]) OR "Sports"[Mesh]) OR "Exercise Therapy"[Mesh]) OR "Walking"[Mesh]) OR "Resistance Training"[Mesh]) OR "Endurance Training"[Mesh] | 41061 |
| #9 | Search: ((((((((((((((((((((((((((((((Exercises[Title/Abstract]) OR (Physical Activity[Title/Abstract])) OR (Activities, Physical[Title/Abstract])) OR (Activity, Physical[Title/Abstract])) OR (Physical Activities[Title/Abstract])) OR (Exercise, Physical[Title/Abstract])) OR (Exercises, Physical[Title/Abstract])) OR (Physical Exercise[Title/Abstract])) OR (Physical Exercises[Title/Abstract])) OR (Acute Exercise[Title/Abstract])) OR (Acute Exercises[Title/Abstract])) OR (Exercise, Acute[Title/Abstract])) OR (Exercises, Acute[Title/Abstract])) OR (Exercise, Isometric[Title/Abstract])) OR (Exercises, Isometric[Title/Abstract])) OR (Isometric Exercises[Title/Abstract])) OR (Isometric Exercise[Title/Abstract])) OR (Exercise, Aerobic[Title/Abstract])) OR (Aerobic Exercise[Title/Abstract])) OR (Aerobic Exercises[Title/Abstract])) OR (Exercises, Aerobic[Title/Abstract])) OR (Exercise Training[Title/Abstract])) OR (Exercise Trainings[Title/Abstract])) OR (Training, Exercise[Title/Abstract])) OR (Trainings, Exercise[Title/Abstract])) OR (strength training[Title/Abstract])) OR (moving[Title/Abstract])) OR (walking[Title/Abstract])) OR (lifting[Title/Abstract])) OR (weights training[Title/Abstract])) OR (resisitance[Title/Abstract]) | 49078 |
| #10 | ((((((((((((((((((((((((((((((Exercises[Title/Abstract]) OR (Physical Activity[Title/Abstract])) OR (Activities, Physical[Title/Abstract])) OR (Activity, Physical[Title/Abstract])) OR (Physical Activities[Title/Abstract])) OR (Exercise, Physical[Title/Abstract])) OR (Exercises, Physical[Title/Abstract])) OR (Physical Exercise[Title/Abstract])) OR (Physical Exercises[Title/Abstract])) OR (Acute Exercise[Title/Abstract])) OR (Acute Exercises[Title/Abstract])) OR (Exercise, Acute[Title/Abstract])) OR (Exercises, Acute[Title/Abstract])) OR (Exercise, Isometric[Title/Abstract])) OR (Exercises, Isometric[Title/Abstract])) OR (Isometric Exercises[Title/Abstract])) OR (Isometric Exercise[Title/Abstract])) OR (Exercise, Aerobic[Title/Abstract])) OR (Aerobic Exercise[Title/Abstract])) OR (Aerobic Exercises[Title/Abstract])) OR (Exercises, Aerobic[Title/Abstract])) OR (Exercise Training[Title/Abstract])) OR (Exercise Trainings[Title/Abstract])) OR (Training, Exercise[Title/Abstract])) OR (Trainings, Exercise[Title/Abstract])) OR (strength training[Title/Abstract])) OR (moving[Title/Abstract])) OR (walking[Title/Abstract])) OR (lifting[Title/Abstract])) OR (weights training[Title/Abstract])) OR (resisitance[Title/Abstract]) OR ((((("Exercise"[Mesh]) OR "Sports"[Mesh]) OR "Exercise Therapy"[Mesh]) OR "Walking"[Mesh]) OR "Resistance Training"[Mesh]) OR "Endurance Training"[Mesh] | 718744 |
| #11 | ((((((((((((((((((((((Qualitative Research[MeSH Terms]) OR (grounded theory[MeSH Terms])) OR (interviews as topic[MeSH Terms])) OR (nursing methodology research[MeSH Terms])) OR (focus groups[MeSH Terms])) OR (Qualitative[Title/Abstract])) OR (grounded theory[Title/Abstract])) OR (interviews as topic[Title/Abstract])) OR (nursing methodology research[Title/Abstract])) OR (focus groups[Title/Abstract])) OR (focus group[Title/Abstract])) OR (interviews[Title/Abstract])) OR (interview[Title/Abstract])) OR (phenomenolog*[Title/Abstract])) OR (ethnograph*[Title/Abstract])) OR (case study[Title/Abstract])) OR (historic research[Title/Abstract])) OR (action research[Title/Abstract])) OR (experience*[Title/Abstract])) OR (view*[Title/Abstract])) OR (perspective*[Title/Abstract])) OR (perception*[Title/Abstract])) OR (attitude*[Title/Abstract]) | 3088508 |
| #12 | #3 AND #6 AND #7 AND #10 AND #11 | 94 |
| Web of science | | |
| #1 | Tumor*OR Neoplasm* OR Cancer* OR Leukemia* OR Oncology* OR "Malignant Neoplasm" OR Malignancy* OR "Malignant Neoplasms" OR "Neoplasm, Malignant" OR "Neoplasms, Malignan" | 3165189 |
| #2 | child* OR Adolescent* OR Teen* OR Youth* | 2341285 |
| #3 | Parent* OR caregiver* OR Family OR father OR mother |  |
| #4 | exercis* OR walk* OR sport* OR train* OR physical activit* OR resistance | 4028940 |
| #5 | Qualitative OR "grounded theory" OR "nursing methodology research" OR focus groups OR focus group OR interview OR interviews OR phenomenolog* OR ethnograph* OR case study OR historic research OR action research OR experience* OR view* OR perspective* OR perception* OR attitude* | 8167653 |
| #6 | #1 AND #2 AND #3 AND #4 AND #5 | 1044 |
| Cochrane |  |  |
| #1 | Tumor*OR Neoplasm* OR Cancer* OR Leukemia* OR Oncology* OR "Malignant Neoplasm" OR Malignancy* OR "Malignant Neoplasms" OR "Neoplasm, Malignant" OR "Neoplasms, Malignan" in Title Abstract Keyword | 219725 |
| #2 | child* OR Adolescent* OR Teen* OR Youth* in Title Abstract Keyword | 292948 |
| #3 | Parent* OR caregiver* OR Family OR father OR mother in Title Abstract Keyword | 124548 |
| #4 | exercis* OR walk* OR sport* OR train* OR physical activit* OR resistance in Title Abstract Keyword | 342463 |
| #5 | Qualitative OR "grounded theory" OR "nursing methodology research" OR focus groups OR focus group OR interview OR interviews OR phenomenolog* OR ethnograph* OR case study OR historic research OR action research OR experience* OR view* OR perspective* OR perception* OR attitude* in Title Abstract Keyword | 390398 |
| #6 | #1 AND #2 AND #3 AND #4 AND #5 | 349 |
| CINAHL |  |  |
| S1 | TI Tumor*OR Neoplasm* OR Cancer* OR Leukemia* OR Oncology* OR "Malignant Neoplasm" OR Malignancy* OR "Malignant Neoplasms" OR "Neoplasm, Malignant" OR "Neoplasms, Malignan" | 397362 |
| S2 | TI child* OR Adolescent* OR Teen* OR Youth* | 453800 |
| S3 | TI Parent* OR caregiver* OR Family OR father OR mother | 191638 |
| S4 | TI exercis* OR walk* OR sport* OR train* OR physical activit* OR resistance | 241275 |
| S5 | TI Qualitative OR "grounded theory" OR "nursing methodology research" OR focus groups OR focus group OR interview OR interviews OR phenomenolog* OR ethnograph* OR case study OR historic research OR action research OR experience* OR view* OR perspective* OR perception* OR attitude* | 416934 |
| S6 | S1 AND S2 AND S3 AND S4 AND S5 | 9 |
| EMBASE |  |  |
| #1 | 'malignant neoplasm'/exp | 4554437 |
| #2 | 'cancer':ab,ti OR 'cancers':ab,ti OR 'malignant neoplasia':ab,ti OR 'malignant neoplastic disease':ab,ti OR 'malignant tumor':ab,ti OR 'malignant tumour':ab,ti OR 'neoplasia, malignant':ab,ti OR 'neoplasmic malignancy':ab,ti OR 'neoplastic malignancy':ab,ti OR 'oncologic malignancy':ab,ti OR 'oncological malignancy':ab,ti OR 'tumor, malignant':ab,ti OR 'tumoral malignancy':ab,ti OR 'tumorous malignancy':ab,ti OR 'tumour, malignant':ab,ti OR 'malignant neoplasm':ab,ti | 3218251 |
| #3 | #1 OR #2 | 5425617 |
| #4 | 'child'/exp OR 'adolescent'/exp | 4327646 |
| #5 | 'child':ab,ti OR 'children':ab,ti OR 'teenager':ab,ti OR 'adolescent':ab,ti | 2108291 |
| #6 | #4 OR #5 | 4759692 |
| #7 | 'parent'/exp | 289293 |
| #8 | 'parent':ab,ti OR 'caregiver':ab,ti OR 'family':ab,ti OR 'father':ab,ti OR 'mother':ab,ti | 1588019 |
| #9 | #7 OR #8 | 1742953 |
| #10 | 'physical activity'/exp OR 'sport'/exp OR 'exercise'/exp | 981297 |
| #11 | 'exercis*':ab,ti OR 'walk*':ab,ti OR 'sport*':ab,ti OR 'train*':ab,ti OR 'physical activit*':ab,ti OR 'resistance*':ab,ti | 2806691 |
| #12 | #10 OR #11 | 3186749 |
| #13 | 'qualitative research'/exp OR 'grounded theory'/exp OR 'interview'/exp | 447624 |
| #14 | qualitative:ab,ti OR 'grounded theory':ab,ti OR 'nursing methodology research':ab,ti OR 'focus groups':ab,ti OR 'focus group':ab,ti OR interview:ab,ti OR interviews:ab,ti OR phenomenolog*:ab,ti OR ethnograph*:ab,ti OR 'case study':ab,ti OR 'historic research':ab,ti OR 'action research':ab,ti OR experience*:ab,ti OR view*:ab,ti OR perspective*:ab,ti OR perception*:ab,ti OR attitude*:ab,ti | 4012764 |
| #15 | #13 OR #14 | 4091511 |
| #16 | #3 AND #6 AND #9 AND #12 AND #15 | 1016 |
| **CNKI** | | |
|  | SU=('肿瘤'+'癌症'+'白血病') AND SU=('儿童'+'患儿'+'青少年') AND SU=('父母'+'照顾者'+'母亲'+'父亲') AND SU=('运动'＋'有氧运动'＋'抗阻训练'＋'阻力训练'＋'体力活动'＋'体育锻炼'＋'身体活动') AND SU=('质性研究'+'扎根理论'+'现象学'+'描述性'+'行动研究'+'历史研究'+'民族志'+'人种学'+'个案研究'+'访谈') | 1 |
| **Wan Fang Data** | | |
|  | 主题:('肿瘤'+'癌症'+'白血病') AND 主题:('儿童'+'患儿'+'青少年') AND 主题:('父母'+'照顾者'+'母亲'+'父亲') AND 主题:('运动'＋'有氧运动'＋'抗阻训练'＋'阻力训练'＋'体力活动'＋'体育锻炼'＋'身体活动') AND 主题:('质性研究'+'扎根理论'+'现象学'+'描述性'+'行动研究'+'历史研究'+'民族志'+'人种学'+'个案研究'+'访谈') | 0 |
| **VIP** | | |
|  | (M=(肿瘤+癌症+白血病) OR R=(肿瘤+癌症+白血病)) AND (M=(儿童+患儿+青少年) OR R=(儿童+患儿+青少年)) AND (M=(父母+照顾者+母亲+父亲) OR R=(父母+照顾者+母亲+父亲)) AND (M=(运动＋有氧运动＋抗阻训练＋阻力练习＋体力活动＋体育锻炼＋步数＋步行距离) OR R=(运动＋有氧运动＋抗阻训练＋阻力练习＋体力活动＋体育锻炼＋步数＋步行距离)) AND (M=(质性研究+扎根理论+现象学+描述性+行动研究+历史研究+民族志+人种学+个案研究+访谈) OR R=(质性研究+扎根理论+现象学+描述性+行动研究+历史研究+民族志+人种学+个案研究+访谈)) | 0 |
