# Supplementary material for: Parents' perceptions of physical activity for their children with cancer: a qualitative meta-synthesis
Source: Front Pediatr. 2025 Mar 28;13:1402516. doi: 10.3389/fped.2025.1402516 (PMC11985796; doi:10.3389/fped.2025.1402516)
Supplement: Supplementary file 2 [file Table2.docx]

**Supplementary File2: ENTREQ Statement**

Enhancing transparency in reporting the synthesis of qualitative research: the ENTREQ statement (Tong et al., 2012).

| **Item** | **Guide and Description** |
| --- | --- |
|  |  |
|  |  |
| **1.Aim** | To synthesize the qualitative evidence and understand the parents' perceptions of physical activity for their children with cancer (**see chapter 1).** |
| **2.Synthesis methodology** | This review was designed using thematic synthesis (**see chapter 2.1).** |
| **3. Approach to searching** | Studies published in English or Chinese were eligible. All studies had to reach the standard accepted by PICOS to be included in this review **(see chapter 2.2).** |
| **4. Inclusion criteria** | P= participant, I= phenomenon of interest, Co= context, and S= type of study **(see chapter 2.2 and Table 1).** |
| **5. Data sources** | We searched Web of Science, PubMed, CINAHL, Embase, Cochrane Library and three Chinese databases (CNKI, Wan Fang Data and VIP) for articles published before October 2023 in either English or Chinese. **(see chapter 2.3).** |
| **6. Electronic search strategy** | The search terms were "Neoplasms", "Tumor", "Cancer", "Child", "Adolescent", "Parent", "Mother", "Exercise", "Physical Activity", "Sports" and "Qualitative Research". Furthermore, the reference lists of the included papers were searched to find all relevant studies. **(see** **Supplementary file 1)** |
| **7. Study screening methods** | The PRISMA flowchart covers the selection process for the inclusion of studies. Endnote 20 reference management software was used to manage the search items. After deleting duplicate records, two reviewers independently screened the study titles and abstracts and then read each study for further evaluation. Disagreements between reviewers regarding eligibility were resolved through discussion with a third reviewer. (**see chapter 2.4).** |
| **8. Study characteristics** | The details of the research characteristics are shown in Table 2  **(see chapter 3.2).** |
| **9. Study selection results** | The results of research selection are shown in Figure 1 (**see chapter 3.1).** |
| **10. Rational for appraisal** | The rationale for the quality assessment was to assess the quality of each study that supported the findings of this systematic review **(see chapter 2.5).** |
| **11. Appraisal items** | The quality of included studies was independently evaluated by two reviewers using the JBI qualitative assessment tool. The list consists of 10 items, each of which is "yes," "no," or "unclear." **(see chapter 2.5).** |
| **12. Appraisal process** | Discrepancies between reviewers were discussed and settled by consulting a third reviewer. The aim of the qualitative assessment was to highlight the quality of the evidence on the subject based on a systematic and standardized process, rather than to exclude poor quality studies. **(see chapter 2.5**). |
| **13. Appraisal results** | The study quality assessment can be seen in Table 3 **(see Table 3).** |
| **14. Data Extraction** | In the first phase, two reviewers independently coded all descriptions related to parents' experiences and perceptions of PA for their children with cancer. In the second phase, reviewers looked for similarities and differences between codes and grouped to create new codes to organize descriptive themes. In the third phase, analytical themes that explained all the descriptive themes as well as inferred experiences and perceptions were generated by repeatedly reviewing and analyzing the descriptive themes from the previous stage. **(see chapter 2.6).** |
| **15. Number of reviewers** | There are three reviewers in total. **(see chapter 2.4) (see chapter 2.5)** |
| **16. Coding** | Comprehensive line-by-line encoding of text to capture context, meaning, and ideas **(see chapter 2.6).** |
| **17. Study Comparison** | The concept of representing the entire dataset was developed after multiple readings of all included studies **(see chapter 2.6)** |
| **18. Derivation of themes** | The approach to developing topics and subtopics is inductive and iterative **(see chapter 2.6).** |
| **19. Quotations** | Citations extracted from primary studies are shown in italics, with quotation marks placed in the body of the findings to support the findings (**see chapter 3.5).** |
| **20.Synthesis output** | The lines of argumentation derived represent parents' understanding and perceptions of their children with cancer's participation in physical activity. These perspectives are critical to promoting physical activity in children with cancer and can help children build a commitment to lifelong physical activity (**see chapters 3, 4 & 5**). |
